# Prevalence of Dementia and Mild Cognitive Impairment in a Rural Area of Sivas, Turkey

**DOI:** 10.7759/cureus.13069

**Published:** 2021-02-02

**Authors:** İlteriş Ahmet Şentürk, Hatice Melek Başar, Gülay Uzun Soykök, Hatice Balaban, Özlem Kayım Yıldız, Ertuğrul Bolayır, Suat Topaktaş

**Affiliations:** 1 Neurology, Bağcılar Education and Training Hospital, Istanbul, TUR; 2 Psychiatry, Beylikdüzü State Hospital, Istanbul, TUR; 3 Neurology, Kayseri City Hospital, Kayseri, TUR; 4 Neurology, Liv Hospital, Ankara, TUR; 5 Neurology, Cumhuriyet University Medical School, Sivas, TUR

**Keywords:** dementia, prevalence, mild cognitive impairment, risk factors

## Abstract

Introduction

The exact prevalence of dementia in Turkey is unknown. The purpose of this study was to determine the frequency of dementia in members of the population aged 60 years and older, as well as the influence of detailed sociodemographic factors on the prevalence rate in Sivas City Center, a large city in the middle of Anatolia, Turkey.

Methods

This was a cross-sectional, simple random sampling, door-to-door, population-based study. A total of 500 individuals aged 60 and older from the city center region of Sivas, Turkey, were involved. A sociodemographic data form, the Standardized Mini-Mental Test, the clock drawing test, the Cornell Scale for Depression in Dementia, and the Daily Life Activities and Instrumental Daily Life Activities tests were used in the screening phase.

Results

A total of 500 individuals ranging in age from 60 to 95 years were assessed. A total of 84 participants were diagnosed with dementia. The dementia prevalence was found to be 16.8% in Sivas city province. Dementia was associated with age (p<0.001), female sex (p<0.001), marital status (p<0.001), family income (p<0.001), and the absence of formal education (p<0.001).

Conclusions

This study is the first community-based study of cognitive impairment in Turkey, with a study design, procedures, and diagnostic criteria designed to determining the rate of dementia. Old age, a higher score on the Cornell Scale for Depression in Dementia, and a low educational level were independent risk factors for dementia. Further studies are required to confirm these results.

## Introduction

As with the global population, Turkey’s population is also growing, and its aging population has increased with improved life expectancy [1]. Low socioeconomic status and education level increase the prevalence of chronic diseases, including dementia [2]. Cognitive impairment and the risk of developing dementia are strongly associated with age [3]. The rapid aging of the population and the resulting increase in the rate of patients with dementia have important implications for health and social care. It has been estimated that 24 million people lived with dementia globally in 2001. In 2010, this figure was approximately 35 million. Dementia is a common, progressive, and disabling neurodegenerative disease, which indicates early cognitive decline and functional disability [4,5]. Alzheimer’s disease (AD) and vascular dementia are the most common types of dementia [6]. The rising prevalence of AD and its associated economic implications have been well documented in developed countries [5]. However, the exact prevalence of the disease in developing countries is not fully known.

The prevalence of dementia varies among different countries in the world and in different geographical regions of the same country. The rate has been reported to be lower in Asian countries than in the United States and Europe. The number of studies on this subject in Turkey is limited [7-9]. All the studies conducted in Turkey reveal dementia prevalence rates similar to those of industrial and developed cities of the world. Sivas, a city in Turkey’s Central Anatolia Region, is a less developed city where farming and animal husbandry are dominant, and its residents have a low level of education.

This study aimed to determine the prevalence of dementia in members of the population aged 60 years and older. This epidemiological study in the city center of Sivas utilized the simple random sampling method. Cognitive screening tests, behavioral scales, and functional scales were used in the study, and dementia risk factors were evaluated in detail.

## Materials and methods

According to the 2008 data from the address-based registration system in the city center, the total population aged 60 years and older was 29,655. The study was conducted between January 2010 and January 2011. In order to have a balanced socioeconomic status in the study, different numbers of elderly people from different neighborhoods were identified. Households were selected from all neighborhoods using the simple random sampling method, and face-to-face interviews were conducted by visiting these households. The households that could not be contacted were visited later, and their records were taken. When a household refused to participate, the immediately adjacent household was included in the study. In total, 500 people aged 60 and older were included in the study.

Evaluations were made via face-to-face interviews. Screening questionnaires as well as depression scales were used. Information was obtained from the individuals and their relatives for the evaluation of daily life activities, and neurological examinations were also performed. Before the evaluation, each individual was informed, and their written consent was obtained.

During the evaluation, individuals who were not with their relatives at the time of the first visit were interviewed again during a second visit when they had relatives with them to provide reliable information. Exclusion criteria included individuals with severe visual and hearing impairments, serious psychiatric disorders such as psychosis, an inability to speak, and an inability to communicate properly. Individuals with the specified conditions and those who did not want to participate in the study were not included in the study.

An ethics committee decision from Cumhuriyet University and the necessary permissions from Sivas Governorship and Sivas Provincial Security Directorate were obtained for the study.

Sociodemographic form

A sociodemographic data form was prepared to obtain sociodemographic data (Table 1). Independent variables included gender, age, marital status, educational level, profession, monthly income, and people with whom the subjects lived, as well as other diseases and medications.

**Table 1:** Sociodemographic status of the participants TL, Turkish Lira

	N (%)
Gender	
Female	260 (52)
Male	240 (48)
Education	
Uneducated	181 (36.2)
Literate	59 (11.8)
Primary school	200 (40)
Secondary school	29 (5.8)
Senior high school	24 (4.8)
University	7 (1.4)
Marital status	
Married	345 (69)
Widowed	154 (30.8)
Unmarried	1 (0.2)
Occupation	
Employee	1(0.2)
Unemployed	272 (54.4)
Pensioner	227 (45.4)
Family income	
None	34 (6.8)
<550 TL	117 (23.4)
550–1500 TL	345 (69)
>1500 TL	4 (0.8)
Family member number	
None	39 (7.8)
One person	272 (54.4)
More than one	189 (37.8)

Standardized Mini-Mental State Examination Test

The Standardized Mini-Mental State Examination Test (SMMSE) was produced with the aim of quantitatively evaluating cognitive performance using standard neuropsychiatric examination methods. In terms of monitoring the course of dementia syndromes and responses to treatment, it is the most popular test used in epidemiological studies in most populations in the research field. It consists of 11 items grouped under five main headings: orientation, recording memory, attention and calculation, recall, and language. An evaluation is made over a total of 30 points [10]. The validity and reliability study of the SMMSE for Turkish society was performed by both educated and uneducated individuals [11]. In SMMSE, a score of 25 and above indicates normal cognitive function. Furthermore, 20-24 points indicate mild cognitive impairment, 15-19 points indicate moderate cognitive impairment, and a score below 15 indicates severe cognitive impairment [10].

Clock drawing test

The clock drawing test (CDT) is accepted as a good screening tool due to features such as its easy application and short application time, the simple scoring, and its high sensitivity and specificity in the diagnosis of dementia. Moreover, the CDT evaluates executive functions and visual-spatial functions, which are commonly used in dementia screening (and which the SMMSE cannot adequately evaluate). It has been reported that using these two tests together is optimum in terms of sensitivity for dementia diagnoses [12]. The validity and reliability study of the CDT in the Turkish population aged 50 years and older was performed by Can et al. [13].

Cornell Scale for Depression in Dementia

The Cornell Scale for Depression in Dementia (CSDD) consists of 19 sub-items collected in five subgroups. These subgroups included items that evaluate mood-related symptoms, behavioral disorders, physical symptoms, cyclic functions, and thought disorders. Each item is graded at three points: a, cannot be evaluated; 0, absent; 1, mild or intermittent; 2, severe [14]. The validity and reliability study of the test in Turkish was performed by Amuk et al. in 2003 [15]. A score of 10 and above on the test suggests probable major depression, whereas a score above 18 corresponds to definite major depression [16].

Activities of Daily Living/Instrumental Activities of Daily Living Scale 

The Activities of Daily Living (ADL)/Instrumental Activities of Daily Living Scale (IADL) consists of two components: The first includes nine items related to self-care, and the second contains seven items related to ADL/IADL. Each item is evaluated on a three-point scale. It is filled out by the patients and their relatives [16].

Diagnosis of dementia

Diagnosis of dementia has been established clinically according to the DSM-4-TR (Diagnostic and Statistical Manual of Mental Disorders, 4th Edition, Text Revision) criteria. Dementia according to DSM-4-TR includes memory impairment, aphasia, apraxia, agnosia, and loss of multiple cognitive areas, including at least one impairment in executive functions. Furthermore, dementia has been referred to as a cognitive impairment that affects professional and social functions and that causes a decrease in a higher pre-existing functional level [17].

Statistical analysis

For statistical analysis, Statistical Package for Social Sciences (SPSS for Windows) Version 16.0 (SPSS Inc., Chicago, IL, USA) was used. The chi-square test was used for the comparison of qualitative data. Spearman correlation analysis was performed for data that did not conform to normal distribution to evaluate the relationship between two variables, and Kendal's tau correlation analysis was conducted to analyze the relationship between ordinal variables. For the comparison of the quantitative data, Student's t-test was used for the parameters displaying normal distribution, and Mann-Whitney’s U test was used to compare the groups that did not conform to normal distribution. The results were evaluated at a 95% confidence interval, and the significance was set at p < 0.05.

## Results

Sociodemographic characteristics

In total, 260 (52%) of the participants were women and 240 (48%) were men. Dementia was detected in 84 (16.8%) of the 500 participants evaluated. The sociodemographic characteristics of the participants in the study are given in Table 1.

Age and gender

The mean age of the participants was 68.5 ± 6.6 years. Furthermore, the mean age of those with dementia was 73.9 ± 8.1 years and that of those without dementia was 67.4 ± 5.7 years. Moreover, 54 (64.3%) of the dementia group were women and 30 (35.7%) were men. The dementia rate was higher in women (p = 0.009).

The mean age of women with dementia was 72.2 ± 7.7, and the mean age of men with dementia was 76.9 ± 8.1. There was a significant difference in terms of age between those with and without dementia (p = 0.001). When the dementia rate was evaluated between groups divided into 10-year age groups, the dementia incidence rate was found to be 8.2% (n = 26) in 63% (315) participants aged 60-69, 23.9% (n = 34) in 28.4% (142) participants aged 70-79, and 55.8% (n = 24) in 8.6% (43) participants aged ≥80. An increase was observed in dementia incidence with increasing age (Table 2).

**Table 2:** Evaluation of the presence and absence of dementia according to age groups p = 0.000

Age groups	Dementia	Dementia	Total
	Yes (%)	No (%)	
60-69	26 (8.2)	289 (91.8)	315
70-79	34 (23.9)	108 (76.1)	142
80 and over	24 (55.8)	19 (44.2)	43

Education level was lower, widowhood status was higher, unemployment rate was higher, and monthly income per person was significantly lower in women than men.

Educational level

Of the 500 participants, 181 (36.2%) were illiterate and 319 (73.8%) were literate. The dementia rate was 26.5% (48) in illiterate participants. Furthermore, 16 (27.1%) were literate but did not go to school, and dementia was detected in 9.04% (18) of primary school graduates, 3.3% (1) of secondary school graduates, and 4.1% of high school graduates. The findings demonstrated that the dementia incidence rate increased as the education level decreased (p = 0.000).

Marital status, family structure, and profession

In terms of marital status, 345 (69%) of the participants were married, 154 (30.8%) were widows, and one was single. Dementia was detected in 11.6% (40) of the married participants and 27.9% (43) of the widowed participants. The dementia rate was found to be significantly higher in widowed participants (p = 0.000).

In total, 3 (7.7%) of the 39 (7.8%) participants who lived alone had dementia, whereas 36 (92.7%) had no dementia. There was also no significant difference among the participants living with one or more individuals in terms of the presence of dementia.

Furthermore, 272 (54.4%) of the 500 participants were unemployed. The majority of the unemployed participants were women, with an unemployment rate of 88.2% (240). Moreover, 32 (11.8%) of the male participants had not worked in a regular job before, 227 (45.4%) were retired, 8.8% of them were women, and 91.2% were men. Cognitive impairment was not detected in any actively working case. In our study, no statistically significant difference was found between occupational groups in terms of dementia. The dementia rate was found to be higher in the unemployed group.

Socioeconomic levels were evaluated according to family monthly incomes

When the monthly income of the participants was examined, results revealed that 10 (29.4%) had no monthly income. Additionally, 23 (19.7%) of 117 participants with a monthly income of less than 550 Turkish Lira (TL) and 51 (14.8%) of 345 individuals with a monthly income of 550-1500 TL had dementia; however, in the group with a monthly income of more than 1500 TL, no dementia was detected (p = 0.01).

Disease status and substance use

A total of 450 (90%) participants had at least one organic disease (Table 3). No significant difference was found between the groups with and without dementia for hypertension, diabetes mellitus (DM), coronary artery disease, hyperlipidemia, cerebrovascular diseases, and depression. The only significant relationship between dementia and chronic systemic diseases was found with chronic obstructive pulmonary disease (COPD). Dementia was found in 26.8% (19) of 71 patients with COPD and in only 15.1% (65) of 429 participants without COPD (p = 0.015). The difference between smoking and alcohol consumption among the various groups was non-significant (Table 3).

**Table 3:** Disease frequency of the participants HT, hypertension; DM, diabetes mellitus; CAD, coronary artery disease; COLD, chronic obstructive lung disease; HL, hypercholesterolemia; BPH, benign prostatic hypertrophy; CVD, cerebrovascular disease

Disease	Number	%
HT	290	58
DM	149	29.8
CAD	121	24.2
COLD	71	14.2
HL	61	12.2
BPH	55	11
Osteoarthritis	30	6
Depression	23	4.6
Thyroid disease	18	3.6
CVD	18	3.6
Cancer	8	1.6
Head trauma	8	1.6
Epilepsy	7	1.4

Evaluation of SMMSE scores

The mean SMMSE score obtained in the study was 26.99 ± 3.20. Table 4 shows the SMMSE scores of the participants. According to SMMSE, there was a positive correlation between education level and CDT and a negative correlation between age and ADL/IADL. The relationships between SMMSE scores, and gender, marital status, and socioeconomic level were evaluated. SMMSE scores were found to be significantly lower in women, unmarried people, and those with low socioeconomic status (p < 0.005).

**Table 4:** SMMSE score distribution of the participants SMMSE, Standardized Mini-Mental State Examination Test

SMMSE score	Number (percent)
Under 15 points (severe cognitive involvement)	3 (0.6)
15–19 points (moderate cognitive involvement)	21 (4.2)
20–24 points (mild cognitive involvement)	68 (13.6)
Over 25 points (normal cognitive state evaluation)	408 (81.6)

Evaluation of CDT 

Average CDT test scores were calculated as 383 ± 2.89. Furthermore, 65.6% (328) of the participants had CDT scores of 5 and below (impairment in the planning and structuring areas of cognition), and in 34.4% (172) participants, CDT scores were found to be 6 or above (normal cognitive examination). Similar to SMMSE, CDT scores in executive and visual spatial abilities of cognition tended to decrease with age. There was a negative correlation between CDT scores and ADL/IADL. The data revealed a decrease in daily and instrumental daily life quality together with a decline in cognitive functions.

Evaluation of the CSDD

When the distribution of participants in our study according to their CSDD scores was examined, 86.6% (434) participants were without depression (0-9 points), 10.4% (52) were with probable major depression, and 2.8% (14) were with definite major depression were identified. The percentage of people with probable major depression was 21.4% (18) in the group with dementia and 8.2% (34) in the group without dementia. The percentage of people with definite major depression was 10.7% (9) in the group with dementia and 1.2% (5) in the non-dementia group, and the difference was statistically significant (Table 5). The results revealed that the comorbidity rate of depression in dementia had increased.

**Table 5:** The incidence of major depression in patients with and without dementia p = 0.001 CSDD, Cornell Scale for Depression in Dementia

	CSDD	CSDD	CSDD
	0–9 score	10–17 score	18 and over
With dementia, N (%)	57 (67.9)	18 (21.4)	9 (10.7)
Without dementia, N (%)	377 (90.6)	34 (8.2)	5 (1.2)

Evaluation of ADL/IADL

The first test evaluated the parameters aimed at providing the basic needs necessary for the survival of life and the second one assessed the parameters for living independently in the society. In our study, the mean ADL score of the participants was 0.60 ± 1.24, and the mean IADL score was 1.08 ± 2.21. In the group with dementia, the mean ADL score was calculated as 2.27 ± 2.02 and the mean IADL score was 4.8 ± 3.17. In the group without dementia, the mean ADL score was calculated as 0.26 ± 0.61 and the mean IADL score was 0.32 ± 0.70.

## Discussion

Dementia and other cognitive problems are common. An estimated 24 million individuals in the world have dementia, and the number affected is expected to double every 20 years [4]. Generally, costs for people with dementia, such as nursing home and long-term care, are prohibitive not only for the health care system but also for families, caregivers, and employers [18].

Detection through door-to-door surveys is the best method in developing countries for early detection of mild cases [19]. This is the first study to report the prevalence of dementia and its risk factors in a community-based catchment area. Dementia prevalence was found to be 16.8% in Sivas City. It was higher than those of similar studies in other regions. It was thought that this difference depends on different sociodemographic characteristics, such as education, sociocultural level, and living standards.

Age is the most important and best-known risk factor for dementia. Studies have demonstrated that both prevalence and incidence of dementia double with every five-year increase in age. In addition, another risk factor is female gender. Female gender has been shown to be associated with an increased risk of dementia, especially at older ages. Similar to previous studies, in our study, women were more likely than men to have dementia. Nevertheless, in our study, female gender was an independent risk factor in the development of dementia. This may be due to the fact that sociodemographic risk factors associated with women are more prominent compared to men, which results in female gender being a risk factor for death from dementia [20].

Results of this study revealed that education reduced dementia risk. The “dose” of education was associated with decreased dementia risk independently of the severity of pathology and also greater brain weight [21]. Harmanci et al.’s study was shown that having a university/college level education is protective against AD [22]. Our data demonstrated a consistent trend of a higher risk of dementia with lower educational level. The relative risks of dementia decreased with increasing educational status.

Studies have suggested an association between marital status and dementia. Although dementia does affect marriage life considerably in married individuals, the risk of dementia is higher in unmarried individuals [23]. In our study, there was a statistically significant difference in the presence of dementia between married and unmarried participants. The prevalence of dementia was higher in non-married participants.

Untreated comorbidities by speeding up the cognitive decline of people with dementia may result in significant financial loss for health and social care systems. Chronic diseases of the brain and mind deserve increased prioritization. Besides disability, they lead to dependency and present stressful, complex, and long-term challenges to carers [24]. As in other studies, our report highlights that while dementia is often viewed as an isolated condition, this patient group suffers from a high prevalence of comorbid medical conditions, with a number of conditions appearing to be “significantly associated” with dementia, including congestive heart failure and DM [25]. However, despite the prevalence of comorbidities among people with dementia, we have found that this group may be receiving poorer levels of care as they suffer a significantly faster decline in daily functioning, a reduced quality of life, and earlier death than people who have the same comorbidities but who do not have dementia.

Neuropsychiatric symptoms, such as apathy, agitation, and depression, are also common. Persons with dementia were twice as likely to have depression as persons without dementia. Among persons with dementia, the prevalence of depression was higher for men than women, and the risk of depression varied by the type of dementia. The study also highlighted that depression was an independent risk factor for the development of dementia, but there was no significant relationship between the presence of depression and the development of dementia.

The SMMSE has been found to be a quick and valuable test for simple bedside screening and for serial assessment of cognitive function in a population. In general, the SMMSE fulfills its goal of providing a brief screening test that quantitatively assesses the severity of cognitive impairment and documents. The SMMSE should not by itself be used as a diagnostic tool to identify dementia. Suggestions for the clinical use of the SMMSE have already been made [26].

The CDT is used for screening for cognitive impairment and dementia and as a measure of spatial dysfunction and neglect. It appears to be a useful adjunct in the assessment and monitoring of the progressive dementias in the community. The CDT meets the defined criteria for a cognitive screening instrument. It taps into a wide range of cognitive abilities, including executive functions. Furthermore, it is quick and easy to administer and score, with excellent acceptability by subjects. Together with informant reports, it can be concluded that the CDT is complementary to the widely used and validated SMMSE and should provide a significant advance in the early detection of dementia and in monitoring cognitive change. A simple scoring system with emphasis on the qualitative aspects of clock drawing should maximize its utility.

In this study, we found a positive correlation between CDT scores and SMMSE scores and a negative correlation between CDT scores, and age, ADL/IADL, and CDDS. These results are in agreement with studies performed with similar methods and comparable populations.

## Conclusions

Dementia presents laboratory, clinical, societal, and economic challenges. This study is the first community-based study of cognitive impairment in Turkey, with a study design, procedures, and diagnostic criteria for dementia. Dementia prevalence was found to be 16.8% in people over 60 years in Sivas city province. Old age, a higher score on the Cornell Scale for Depression in Dementia, and low education level were independent risk factors for dementia. The study found that a devastating disease such as dementia was not well recognized or was neglected in society. Improvement of prevention strategies and caring for people with dementia should be undertaken. Furthermore, strategies for enhancing early identification, treatment, and rehabilitation should be developed for people with dementia. Further studies are required to confirm these results.
